# Estimated incidence of disruptions to event-free survival from non-metastatic cancers in New South Wales, Australia - a population-wide epidemiological study of linked cancer registry and treatment data

**DOI:** 10.3389/fonc.2024.1338754

**Published:** 2024-08-21

**Authors:** Stephen Morrell, David Roder, David Currow, Alexander Engel, Elizabeth Hovey, Craig R. Lewis, Winston Liauw, Jarad M. Martin, Manish Patel, Stephen R. Thompson, Tracey O’Brien

**Affiliations:** ^1^ Division of Cancer Services and Information, Cancer Institute NSW, St Leonards, NSW, Australia; ^2^ Cancer Epidemiology and Population Health, University of South Australia, Adelaide, SA, Australia; ^3^ Faculty of Science, Medicine and Health, University of Wollongong, Wollongong, NSW, Australia; ^4^ Faculty of Medicine and Health, University of Sydney, Sydney, NSW, Australia; ^5^ Northern Sydney Cancer Centre, Royal North Shore Hospital, St Leonards, NSW, Australia; ^6^ Department of Medical Oncology, Prince of Wales Hospital, Randwick, NSW, Australia; ^7^ School of Clinical Medicine, Faculty of Medicine and Health, University of New South Wales, Kensington, NSW, Australia; ^8^ Peritonectomy and Liver Cancer Unit, St George Hospital, Kogarah, NSW, Australia; ^9^ School of Medicine and Public Health, University of Newcastle, Newcastle, NSW, Australia; ^10^ Department of Radiation Oncology, Calvary Mater Hospital Newcastle, Newcastle, NSW, Australia; ^11^ GenesisCare Maitland, Maitland, NSW, Australia; ^12^ Western Clinical School, Faculty of Medicine and Health, University of Sydney, Sydney, NSW, Australia; ^13^ Faculty of Health Sciences, Macquarie University, North Ryde, NSW, Australia; ^14^ Nelune Comprehensive Cancer Centre, Prince of Wales Hospital, Randwick, NSW, Australia; ^15^ Cancer Institute NSW, St Leonards, NSW, Australia

**Keywords:** cancer, recurrence and other disruptive events, survival, breast, colorectal, lung, prostate, melanoma

## Abstract

**Introduction:**

Population cancer registries record primary cancer incidence, mortality and survival for whole populations, but not more timely outcomes such as cancer recurrence, secondary cancers or other complications that disrupt event-free survival. Nonetheless, indirect evidence may be inferred from treatment data to provide indicators of recurrence and like events, which can facilitate earlier assessment of care outcomes. The present study aims to infer such evidence by applying algorithms to linked cancer registry and treatment data obtained from hospitals and universal health insurance claims applicable to the New South Wales (NSW) population of Australia.

**Materials and methods:**

Primary invasive cancers from the NSW Cancer Registry (NSWCR), diagnosed in 2001–2018 with localized or regionalized summary stage, were linked to treatment data for five common Australian cancers: breast, colon/rectum, lung, prostate, and skin (melanomas). Clinicians specializing in each cancer type provided guidance on expected treatment pathways and departures to indicate remission and subsequent recurrence or other disruptive events. A sample survey of patients and clinicians served to test initial population-wide results. Following consequent refinement of the algorithms, estimates of recurrence and like events were generated. Their plausibility was assessed by their correspondence with expected outcomes by tumor type and summary stage at diagnosis and by their associations with cancer survival.

**Results:**

Kaplan-Meier product limit estimates indicated that 5–year cumulative probabilities of recurrence and other disruptive events were lower, and median times to these events longer, for those staged as localized rather than regionalized. For localized and regionalized cancers respectively, these were: breast - 7% (866 days) and 34% (570 days); colon/rectum - 15% (732 days) and 25% (641 days); lung - 46% (552 days) and 66% (404 days); melanoma - 11% (893 days) and 38% (611 days); and prostate - 14% (742 days) and 39% (478 days). Cases with markers for these events had poorer longer-term survival.

**Conclusions:**

These population-wide estimates of recurrence and like events are approximations only. Absent more direct measures, they nonetheless may inform service planning by indicating population or treatment sub-groups at increased risk of recurrence and like events sooner than waiting for deaths to occur.

## 1 Introduction

Cancer recurrences, complications and other disruptive events are key outcome used in clinical practice, trials, and other studies. They have the advantage of enabling earlier assessment of therapeutic benefit by avoiding the need to wait for mortality evidence to accumulate. Ideally, population-based cancer registries would include these outcomes so that health administrations may better monitor outcomes across the population, both to evaluate research translation, and identify differences in emerging outcomes between population sub-groups. Population-based registries rarely record recurrence, cancer progression beyond definitive treatment or other disruptive events, however, largely because of poor population-wide access to the required clinical, imaging, biochemical and other evidence. In addition, difficulties exist determining at a population level whether patients are cancer-free after treatment and thereby at risk of recurrence and like events.

With the advent of new diagnostic and information technologies, means of collecting data on recurrence and other disruptive events population-wide for routine monitoring purposes are improving, but formidable logistical and ethical challenges remain. If achieved, such data would enable earlier evaluation of the translational benefits from novel therapies and their cost-effectiveness. Inequities in these outcomes may be indicated with important health planning implications for targeting supportive and other services. Ongoing population-wide availability of these data would be an important metric to indicate whether inequalities are persisting, and if so, which population sub-groups are most affected. Local health authorities could target their support services accordingly.

In individual patient care, precise clinical measures of recurrence and other disruptive events are critical. They are less so for profiling risk at a population level, or by population sub-group, where an ordinal ranking of risk often would be sufficient. Key questions in this study are whether population-wide markers of recurrence and other disruptive events drawn from our linked (but limited) data can differentiate and predict risk and whether they are associated with survival on an ordinal scale.

Other researchers have focused on development of recurrence indicators at a population level by applying algorithms to the data available to them through population registries, the health system, and health insurance databases. Examples exist in the United States (US) (1–11), United Kingdom (UK) (12, 13), Canada (14–20), Denmark (21, 22), and other Australian settings (23). Attempts to validate these indicators through clinical chart review have generally indicated a high level of accuracy for breast and lung cancers. The purpose of these studies was generally prognostic estimation for clinical purposes, however, rather than mapping variations in risk more broadly at a population level for health-service targeting and evaluation.

Other population estimates of cancer recurrence have come from clinical cohorts that were generally not population-wide and where generalizations to a population level were questionable. Such cohorts mostly were assembled to address specific clinical questions for which they had a tailored study designs, rather than informing monitoring and evaluation of cancer outcomes in whole populations. Examples include studies in Canada (24), Italy (25, 26), UK (27), Sweden (28), Denmark (29) US (30, 31), Netherlands (32), as well as results of international cohorts (33), and systematic reviews (34).

To collect population-wide data on recurrence and other disruptive events data in Australian, we used the New South Wales Cancer Registry (NSWCR). This is the largest population-based cancer registry in Australia, covering the State of NSW, which includes about a third of the Australian population. NSWCR data were used as the linkage spine to which person-level data were linked from: hospital admissions; emergency departments; universal health insurance claims from the Medical Benefits Schedule (MBS) and Pharmaceutical Benefits Scheme (PBS); and mortality records. Extensive safeguards were used to protect patient privacy while retaining scope for detailed data analyses along the diagnosis-treatment-outcome pathway.

We used these data to develop cancer-specific algorithms to generate population-level markers of cancer recurrence and other disruptive events. From the outset, it is acknowledged that these algorithms, although extensively informed by expert clinician guidance for each cancer type, were limited by the sub-optimal range of data available at a whole population level, as applying for example to imaging or pathology testing of recurrences and like events. The key question is whether these algorithmically derived markers nonetheless provided plausible indicators of rates of recurrence and other disruptive events by cancer type and diagnostic stage, and thus were potentially useful as valid indicators. An important question is whether these markers align with expected differences in cancer survival by cancer type and stage.

As a consequence of these data limitations, otherwise unrecorded disruptions that do not constitute a true cancer recurrence potentially can produce inflated recurrence estimates. One likely disruption is suspension of treatments for an extended period due to patient intolerance or wishes followed by treatment resumption that indicates a “recurrence” following the period of *faux* remission. Conversely, without accompanying prognostic information, termination of treatment can also falsely indicate remission when a recurrence (with progression) has occurred when subsequent treatment or palliation indicators are absent. Other disruptive or like events would be extended treatment lacunae interrupted by treatment for a cancer that has shown no signs of remission. Prostate cancer is the exemplar for this scenario. Accordingly, for this paper we use extended terms like ‘cancer recurrence or other disruptive/like events’, and when ‘cancer recurrence’ alone is used it should be regarded as an abbreviation of the longer more accurate expression.

## 2 Materials and methods

### 2.1 Chronicle and genesis of recurrence project

**Figure 1:**
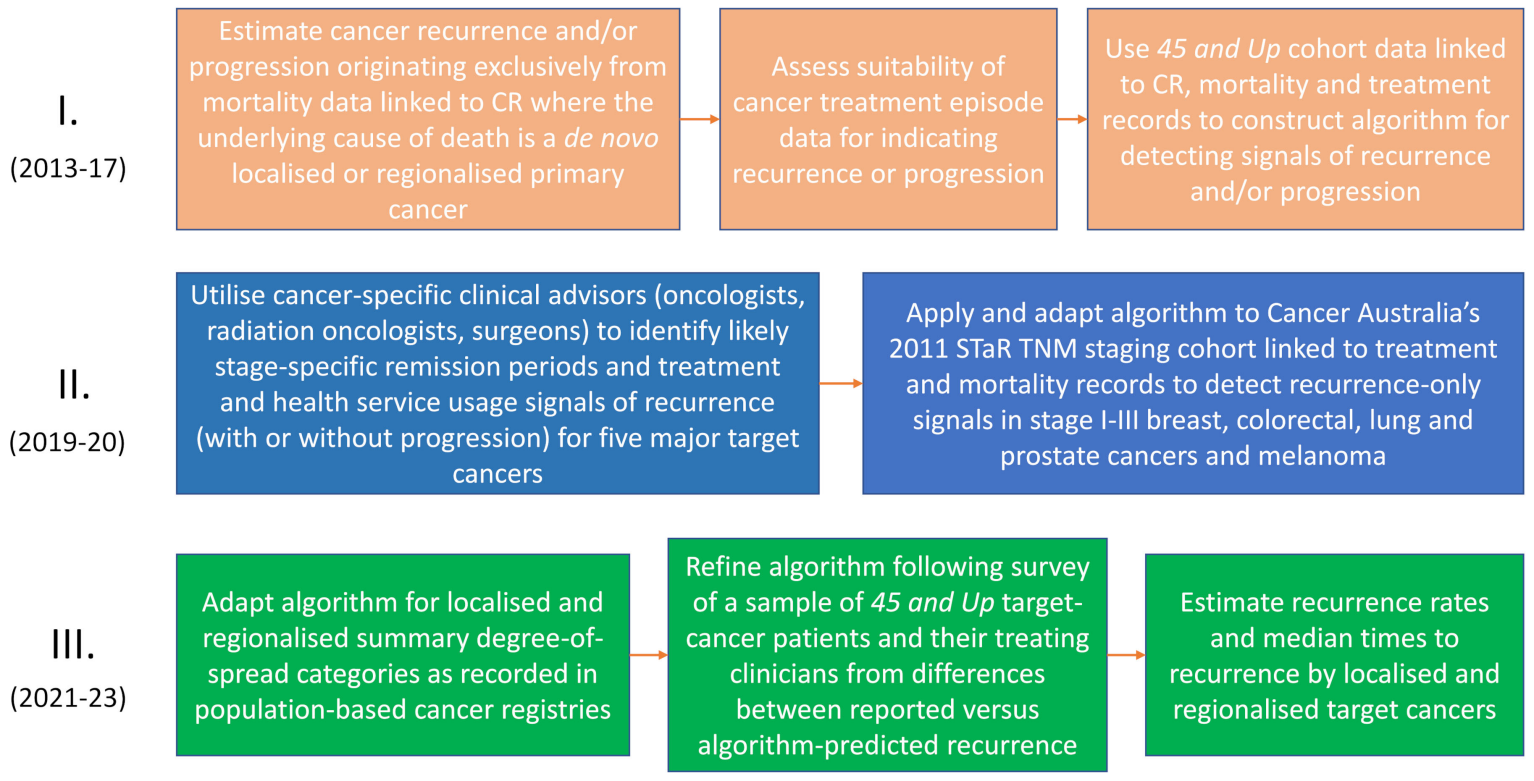
Timeline and evolution of recurrence and like event algorithm development.

The development of algorithms to produce indicators of recurrence and other disruptive events occurred in several phases (See **Figure 1**). Prior to routine linkage of NSW-wide cancer records to health-service diagnostic and treatment data, which became routine from 2018, we used linked data from the *45 and Up* survey to develop initial versions of the algorithms. The use of *45 and Up* cohort participants was opportunistic. They had been recruited from 2006 (n≈267,000), and had given consent for their cancer-registry data to be linked at person level to health- service data.

A significant drawback was the limited number of first-ever cancers diagnosed after recruitment (n≈35,000) to the *45 and Up* study. Some validation of the initial algorithms was possible, primarily using cancer registration data and accompanying treatment episode data partially recorded in the NSW Cancer Registry (NSWCR) which often included systemic and radiotherapy treatments. Accompanying laboratory or pathology test results (e.g., for a secondary cancer) were also provided that may have indicated whether recurrence or progression was present. As not all these ancillary data were retained by the NSWCR, broader algorithm validation in this earlier study was heavily reliant on available systemic therapy data.

In 2019, impetus for further algorithmic development came from Cancer Australia’s nationwide Staging, Treatments and Recurrence (STaR) project, which included $100,000 (AUD) funding support from Cancer Australia to the Cancer Institute NSW for this purpose. A component part of the STaR project involved assigning TNM (Tumor: size and if nearby tissue invaded; Nodes: regional lymph node involvement; and distant Metastases) (35) stage at diagnosis to cohorts of cutaneous melanomas and cancers of the lung, prostate, breast, and colon/rectum diagnosed in 2011 and recorded on cancer registries Australia-wide. Australian population-based cancer registries had not reported routinely on TNM cancer staging previously, although the NSWCR had long reported degree-of-spread (akin to SEER summary stage). While degree of spread had been useful in epidemiological studies, it had limited detail for clinical research. The STaR project assignment of TNM (4 main categories) was more detailed, enabling cancer- and stage-specific variability in treatment pathways to be further explored. This process was informed by specialist clinical advisors comprising surgeons, medical and radiation oncologists with sub-specialty expertise in the relevant cancer types.

**Figure 2:**
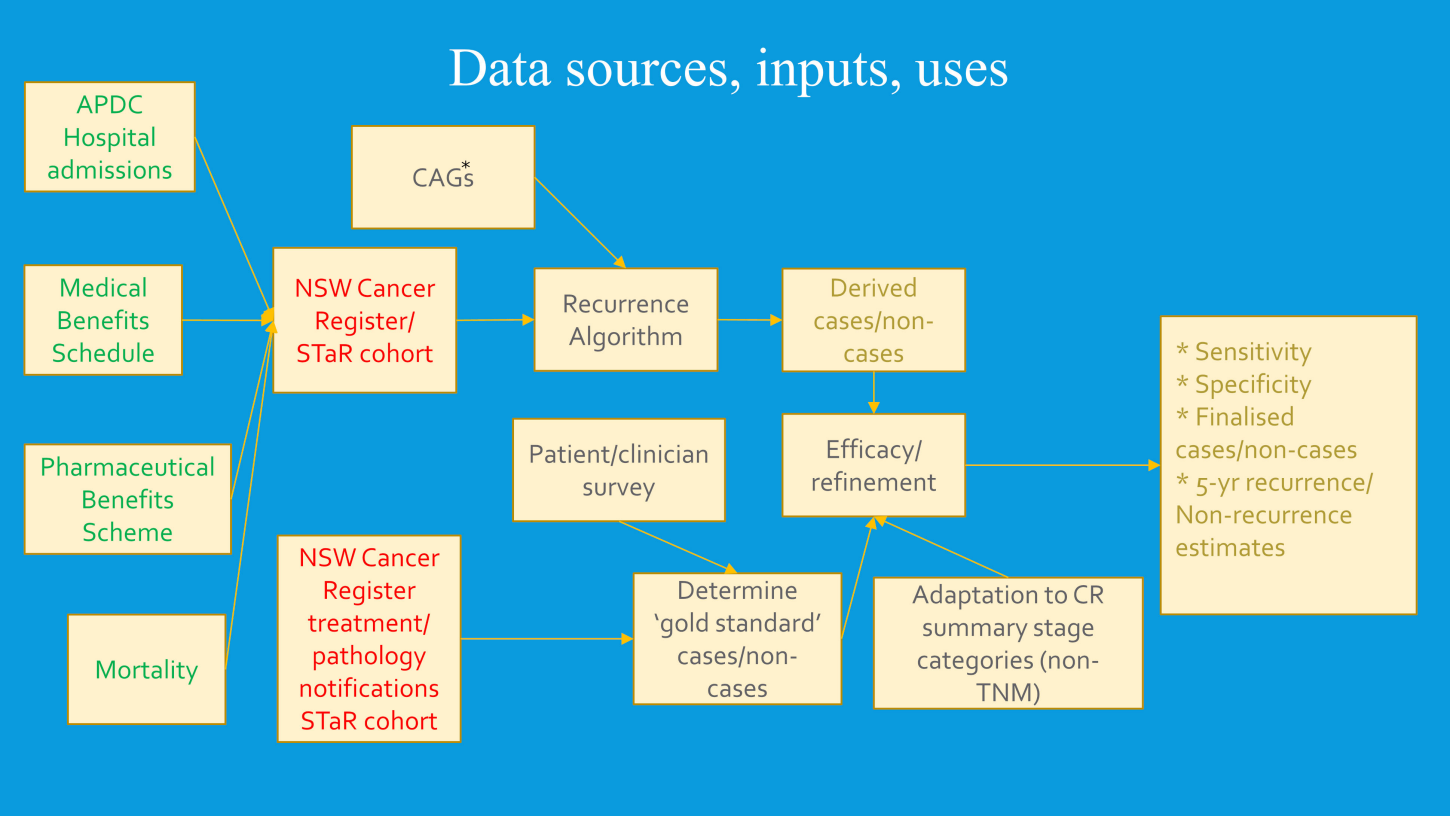
Inputs to recurrence and like event algorithm development. *CAG, Clinical advisory group.

Cancer-specific treatment patterns and scenarios expected by clinicians were incorporated into algorithm development. Informed by the clinical advice, we set expected periods and intervals following cancer diagnosis for definitive treatment. This varied with cancer site, stage and type of definitive treatment, and the collective judgement of the clinical advisors. It guided our decisions as to whether breaks (gaps) in the treatment-data continuum likely reflected cancer remissions or temporary cessations for other reasons. Detection of recurrence-related events in this study was reliant on identification of likely remissions and deciding whether subsequent treatments were likely to be a continuing part of the primary round of care or treatment of recurrences or like events. A broad outline of the algorithm development and data sources used is shown in **Figure 2**, and its operationalization in section 2.5.

Our initial recurrence estimates from the STaR project were broadly consistent with expectations of recurrence rates from the literature, but artefacts in Kaplan-Meier time-to-recurrence step functions suggested further investigation. To this end, the Cancer Institute NSW commissioned the Sax Institute (the agency responsible for administering the *45 and Up* study) to survey a random sample of this cancer cohort and their treating clinicians. Initial application of the STaR-based algorithm had classified cancers by TNM category at diagnosis as having a recurrence (or like event) or being event free. This classification was revisited using the additional patient and clinician reporting gained through the survey. While these reports proved useful, they were imperfect. As an example, in some cases clinicians and patients equated cancer recurrences with progression. On other occasions, recurrence reports were questionable due to the short time elapsing following adjuvant treatment. Nonetheless, the survey data were a useful guide, which after comparison with the linked treatment records for each mismatch, enabled refinements to be made to the algorithm. For instance, a strong indicator of lung cancer recurrence was found to be subsequent CT-directed needle lung biopsies or thoracic paracentesis, so these procedures were added to the algorithm to increase accuracy.

Following these developments, the “improved” algorithms were applied to all diagnoses for the five cancer groupings across NSW from 2001 to 2018. We now report on recurrence and like disruptive events using these indicators. In particular, we investigate their construct validity, as indicated by associations with cancer type, diagnostic stage (localized or regional), and cancer-specific survival. We also compared our indicators of recurrence and other disruptive events with recurrence rates reported in the literature and based on validated algorithms.

### 2.2 Cancer cohort and data management

All single-only primary cancer diagnoses, recorded by the NSWCR from July 2001 to December 2018 for the five selected common cancers, were included. The cohort was linked to diagnostic and treatment data held by NSW hospital records; universal health-insurance data (medical and pharmaceutical) held nationally; and mortality data recorded by the NSW Registry of Births, Deaths and Marriages, and equivalent mortality data collected in other Australian states. These deaths were classified by underlying cause as cancer or non-cancer deaths. Cohort data were linked using probabilistic linkage by the NSW Centre for Health Record Linkage for treatment databases located in NSW (hospital admissions and mortality), and by the Australian Institute of Health and Welfare for databases located nationally (MBS and PBS). The latest date of primary cancer diagnosis in this study was 18 December 2018, which was defined as the censoring date for study follow-up in the absence of earlier death. Due to the latest date of follow-up in the linked non-cancer treatment/outcome data sources extending to 2022, the possibility existed that some of these treatment/outcome events would refer to one or more additional primary cancers diagnosed after the 31 December 2018 cancer diagnosis cut off and thus not recorded in the NSWCR data used for this study. Consequently, to prevent misclassification as recurrent (unrecorded) primary cancers receiving normal treatment, all cancers classified as recurrent after 31 December 2018 (the latest date of primary cancer diagnosis used in this study), were also re-classified as non-recurrent.

The data were managed so as to minimize risk to privacy (36). Analyses were undertaken in a high-security curated research environment isolated from the internet called SURE (Secure Unified Research Environment). SURE is operated under the auspices of an independent agency, the Sax Institute (37).

### 2.3 Definition of cancer recurrence and like events

Markers of recurrence and other events disrupting event-free survival were classified as positive when they occurred following an extended period of absence of recorded treatment subsequent to a prior treatment episode (i.e., an extended period interpreted as a remission). The duration of the “extended period” differed by cancer type and diagnostic stage. Twelve months generally qualified as the minimum period of clinical absence for Stage I (localized) cancers, while six or nine months were more commonly threshold minimums for Stages II/III (regional spread to adjacent tissue and/or lymph nodes) cancers. These summary stages conform with standard definitions developed by the Surveillance, Epidemiology and End Results Program of the National Cancer Institute (USA) and have been used by cancer registries internationally (38). Specialist clinical advice was used in setting these minimum standards by cancer type. As well, a marker of recurrence (with progression) was assumed if following a period of remission, a secondary cancer was diagnosed. As the NSWCR records primary cancers, these secondary cancer diagnoses in our cohort were regarded as relating to the primary cancers and were detected from a hospital admission recording this diagnosis. Another indication of recurrence was drawn from palliative care. The date of first treatment (whether of curative or palliative intent), or the secondary cancer diagnosis following the period of clinical absence, was taken to be the date of recurrence or occasion of a like disruptive event.

### 2.4 Clinical consultations

These were conducted by clinician teams, each specialized in one of the selected cancer types. Initially, using information available through the linked NSWCR, time distributions and Kaplan-Meier times to recurrence and like disruptive events were calculated for each cancer type by stage. Advice was then obtained from specialist advisers regarding interpretation of the treatment data patterns as to whether they likely were a part of different treatment scenarios or indicative of recurrence and like events. As the data were often limited to the fact of a test event or a treatment procedure, assessments were regarded as indicative of recurrences and like events rather than definitive.

Deliberations of these clinical advisory meetings were recorded and synthesized into operational study rules based on the clinical consensus for each cancer type and stage at diagnosis (**Table 1**). These rules were incorporated into the algorithms to generate markers of recurrence and like events. These markers were specific by cancer type and diagnostic stage, and reflected the treatment pathways considered likely by the clinician advisors.

**Table 1:** Clinical advisory group preliminary considerations and main indicators of recurrence or like events.

Cancer	Stage	Recurrence indicators and considerations
Breast	I	• Non-endocrine systemic therapy after 12 months from diagnosis and three months elapsing following any other treatment • Non-endocrine systemic therapy or mastectomy after 12 months from diagnosis following radiotherapy • Toxicity from systemic therapy can cause gaps of 5 weeks and longer • Interruptions to radiotherapy are rare • Repeat radiotherapy, usually for a different site, after initial radiotherapy • Endocrine therapy is ongoing and changes to endocrine therapy are common • Going from endocrine to non-endocrine systemic therapy indicates recurrence • Re-excision may occur within initial surgery due to inadequate margins at first surgery • Mastectomy following radiotherapy is an indicator of recurrence • Absence of receptor data in routine data
	II/III	• Non-endocrine systemic therapy after 15 months from diagnosis or three months elapsing following any other treatment
	IV	• Response to radio or systemic therapy, as shown by cancer growth indicators or markers (which aren’t recorded in the routine data)
Colorectal	I–III	• Very few Stage I or II cancers recur in first 12 months following treatment • Chemotherapy less common for Stage II (b & c) in 2011 than today • Second course radiotherapy is a strong indicator of recurrence (with 3+ month gap) • Useful to examine colon and rectal cancers separately
Lung	I	• Most stage I surgeries occur without chemotherapy excepting large tumors >4cm which have chemotherapy within 6–8 weeks of surgery
	II	• N0 possible adjuvant chemotherapy; N1 adjuvant therapy • Adjuvant chemotherapy>3months following surgery indicates recurrence • Radiotherapy >6 months from surgery indicates recurrence
	III	• 3–4 months between concurrent radio/chemotherapy indicates recurrence
Melanoma	I–III	• Adjuvant radiotherapy usually occurs within 3 months of diagnosis and 8–12 weeks after surgery • Chemotherapy received in outpatient setting therefore PBS should capture most chemotherapy • Immunotherapy, for treating Stage III cancer, was rare in 2011 • Any treatment administered after 6–12 months of definitive treatment is deemed to be for recurrence • Initial diagnostic excision is followed by definitive excision, supposedly within 90 days, but often later • A third excision 6 months or longer after the second incision is deemed a recurrence • Multiple lesions at presentation or within 12 months of initial presentation are generally ‘secondary’ lesions (part of the same primary cancer, treated serially to maximize MBS reimbursement)
Prostate	I–IV	• Treatment can extend from 5 to 15 years and treatment patterns are complex • Re-excision due to insufficient margins is part of existing treatment • Radiotherapy occurring 6 months or longer following surgery is a recurrence indicator • Later surgery with or without lymph node excision/dissection is considered part of primary treatment • Surgery, high intensity focused ultrasound (HIFU), brachytherapy or cryotherapy 12 months or longer following definitive radiotherapy indicates recurrence • Hormone therapy is a proxy for recurrence (Stage II+) • Watchful waiting and active surveillance is considered part of primary treatment • Incidental findings from watchful waiting/active surveillance may inflate ‘biochemical’ (elevated PSA) recurrence

As an example, for Stage I (localized) breast cancer, a recurrence would be indicated by: non-endocrine systemic therapy occurring after 12 months from diagnosis following a gap of three or more months of no other treatment; or a change from endocrine therapy to non-endocrine systemic therapy (12 or more months following diagnosis); or mastectomy following radiotherapy. Algorithms were further refined by a survey of *45 and Up* participants with cancer and/or their treating clinicians, as reported above (Section 2.1). Operationalization of the algorithm is described below.

### 2.5 Operationalization of algorithms for recurrence and like events

1. Select a cohort of all cases of single-only localized or regionalized primary malignancies from the cancer registry;
2. Link these cancer cases to hospital admissions data to:
  (a) Exclude those with a primary or secondary cancer recorded in a hospital diagnosis with no corresponding record in the NSWCR;
  (b) Exclude those with a hospital diagnosis of a new, different primary cancer differing from the index cancer that post-dates the latest NSWCR cancer diagnosis;
  (c) Flag cases where a secondary cancer diagnosis (C77-C79) or a cancer histology code indicating SNOMED metastasis behavior code (/6) was recorded, and extract hospital episode start date as a candidate earliest date of progression, recurrence, or occurrence-like event;
  (d) Extract hospital episodes and dates of cancer treatment including surgery, radiotherapy, chemotherapy specific to each cancer, and multi-disciplinary team items and palliative care episodes where the primary cancer is also listed in diagnosis codes;
  (e) Classify treatment episodes according to interval between date of cancer diagnosis and date of treatment as definitive treatment(s) or adjuvant treatment(s) specific to each cancer;
  (f) Classify all relevant treatment episodes within broad cancer treatment type and assemble ordered dates of occurrence (as an array) for each;
  (g) For each patient, consolidate these arrays onto a single patient record;
3. From MBS data:
  (a) Extract all cancer-related treatment episodes including surgery, radiotherapy, systemic therapy, multi-disciplinary team items and palliative care items;
  (b) Classify treatments into clinically relevant broad categories and create arrays of ordered treatment episode dates for each category;
  (c) Flag treatments to distinguish these from duplications that may appear in hospital treatment items;
  (d) For each patient, consolidate these arrays onto a single patient record;
4. Extract from PBS items relevant to cancer treatment (ATC root codes L01-L04) to:
  (a) Classify systemic therapies into clinically relevant broad categories and create arrays of ordered treatment episode dates for each category;
  (b) For each patient, consolidate these arrays onto a single patient record;
5. From cancer records, flag those who died of the primary cancer, and link cancer records to death registrations to flag mortality where the underlying cause of death was recorded as the index primary cancer or as a secondary cancer; most of these latter were re-classified as the original primary cancer cause of death by the NSWCR;
6. Link the consolidated cancer, hospital admission, PBS, MBS and mortality records:
  (a) Create arrays combining the hospital admission and MBS treatment episode dates by the broad treatment categories and sort dates within each array in chronological order;
  (b) Use summary-stage and cancer-specific expected treatment(s) combined with likely non-treatment time intervals to classify treatment episodes, and corresponding treatment dates, as recurrence and recurrence-like signals for each patient;
  (c) Select earliest date of treatment from the treatment dates indicating a recurrence and like event and assign as the date of the event.

### 2.6 Statistical analysis

Kaplan-Meier plots of time to recurrence (or like disruptive events), and cancer cause-specific survival analyses, were conducted, with the main comparison strata being: (1) localized and regional diagnostic stage for time to recurrence or like event; and (2) by recurrent or like event versus non-event cases for cancer-specific survival. Strata differences in time to events were assessed by log-rank statistics. Median times to recurrence or like events, in those so affected, were estimated for each cancer by localized and regional diagnostic stage. Times to recurrence or like events were fitted with empirical kernel functions to indicate and compare these time distributions (39, 40), including differences in peaks between diagnostic stages (localized and regionalized).

### 2.7 Ethics approval

Ethical approval for this study was obtained from the NSW Population & Health Services Research Ethics Committee, approval # AU RED: HREC/14/CIPHS/31. Informed consent was obtained from a sample of cancer patients and/or their treating clinicians to participate in a survey of cancer recurrence.

## 3 Results

### 3.1 Rates of recurrence and like events

#### 3.1.1 Breast cancer

Of 36,102 localized cancers 8, 3,954 (11%) were estimated to experience a recurrence or like event (**Table 2**). The cumulative incidence of these events over five years was 6.6%. The median time to 5-year recurrence or like event was 866 days. For regionalized breast cancers (n=1,870), 695 (37%) were estimated to experience such an event. The 5-year cumulative incidence of recurrence or a like event was 34%, with a median time to the event of 570 days.

**Table 2:** Numbers and proportions of recurrence or like events, and 5-year cumulative incidences and median times of 5-year cancer recurrence or like event in those with such indicators, major cancers diagnosed July 2001–December 2018, NSW, Australia.

Cancer	Summary Degree of Spread	Median follow-up time (days)	Recurrences				5-year Recurrence	
			All		5-years		Cumulative incidence	Median time (days)
			n	%	n	%	% (95% CI)	
Breast	Localized (n=36,102)	2,682	3,954	11.0	2,138	5.9	6.6 (6.3–6.9)	866
	Regionalized (n=1,870)	1,585	695	37.2	570	30.5	34.3 (32.0-36.6)	570
	Total (n=37,972)	2,626	4,649	12.2	2,708	7.1	7.9 (7.6–8.2)	855
Colorectal	Localized (n=19,093)	2,147	3,299	17.3	2,345	12.3	14.5 (13.9–15.0)	732
	Regionalized (n=10,364)	1,801	2,815	27.2	2,273	21.9	25.4 (24.5–26.3)	641
	Total (n=29,457)	2,009	6,114	20.8	4,618	15.7	18.3 (17.8–18.8)	692
Lung	Localized (n=8,398)	599	2,809	33.5	2,421	28.8	46.2 (44.9–47.6)	552
	Regionalized (n=2,555)	326	1,143	44.7	1,058	41.4	65.5 (63.1–67.9)	404
	Total (n=10,953)	517	3,952	36.1	3,479	31.8	50.5 (49.3–51.7)	509
Melanoma	Localized (n=42,015)	2,467	6,826	16.3	4,126	9.8	11.1 (10.8–11.5)	893
	Regionalized (n=1,851)	1,172	683	36.9	585	31.6	38.4 (35.9–41.0)	611
	Total (n=43,866)	2,412	7,509	17.1	4,711	10.7	14.0 (13.7–14.4)	853
Prostate	Localized (n=42,688)	2,584	8,294	19.4	5,444	12.8	14.0 (13.7–14.4)	742
	Regionalized (n=8,770)	1,414	3,591	41.0	3,155	36.0	38.7 (37.6–39.8)	478
	Total (n=51,458)	2,351	11,885	23.1	8,599	16.7	18.2 (17.8–18.6)	633

#### 3.1.2 Colorectal cancer

Of 19,093 localized colorectal cancers, 3,299 (17%) were estimated to experience a recurrence or like event. The 5-year cumulative incidence of recurrence or like event was 15% (median time 732 days). For regionalized colorectal cancers (n=10,364), 2,815 (27%) were estimated to experience such an event, with a cumulative 5-year incidence of recurrence or like event of 25%. The median time to the 5-year event was 641 days.

#### 3.1.3 Lung cancer

Of 8,398 localized lung cancers, 2,809 (34%) were estimated to have a recurrence or like event, with a 5-year cumulative incidence of such an event of 46% (median time 552 days). For regionalized cancers (n=2,555), 1,143 (45%) were estimated to have a recurrence or like event, with a 5-year a cumulative incidence of recurrence or like event of 66% and a median time to 5-year event of 404 days.

#### 3.1.4 Skin cancer

Of 42,015 localized melanomas, 6,826 (16%) were estimated to experience a recurrence or like event, with a 5-year cumulative incidence of such an event of 11% (median time 893 days). For regionalized melanoma (n=1,851), 683 (37%) were estimated to have a recurrence or like event, with a cumulative incidence of recurrence or like event at five years of 38% (median time 611 days).

#### 3.1.5 Prostate cancer

Of 42,688 localized prostate cancers, 8,294 (19%) were estimated to experience a recurrence or like event, with a 5-year cumulative incidence of recurrence or like event of 14% (median time 742 days). For regionalized cancers (n=8,770), recurrences or like event were indicated for 3,591 (41%), with a 5-year cumulative incidence of recurrence or like event of 39% (median time 478 days).

### 3.2 Times to recurrence or like event

**Figure 3:**
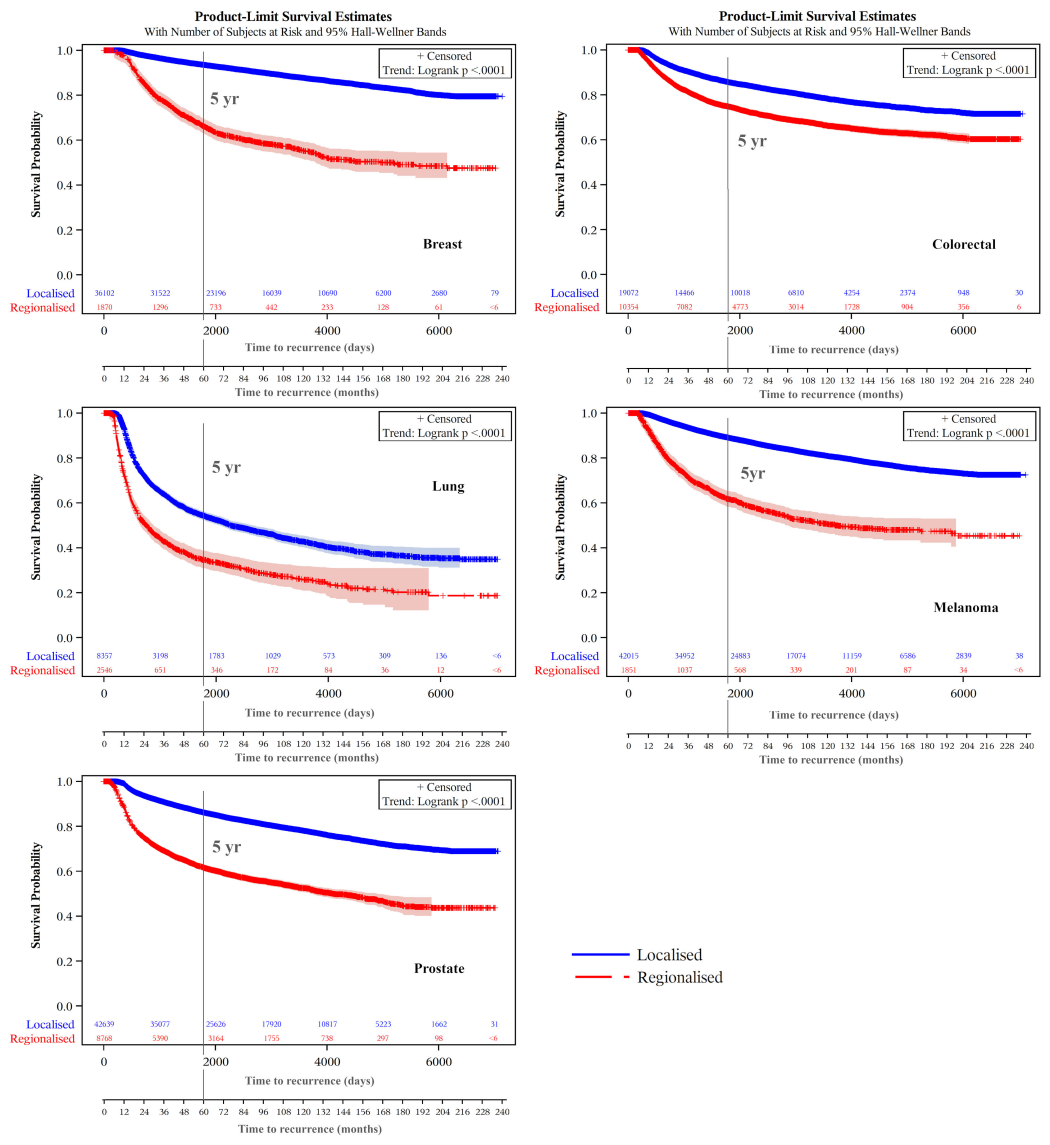
Recurrence and like event time-to-event plots by extent of disease/summary degree of spread.

**Figure 4:**
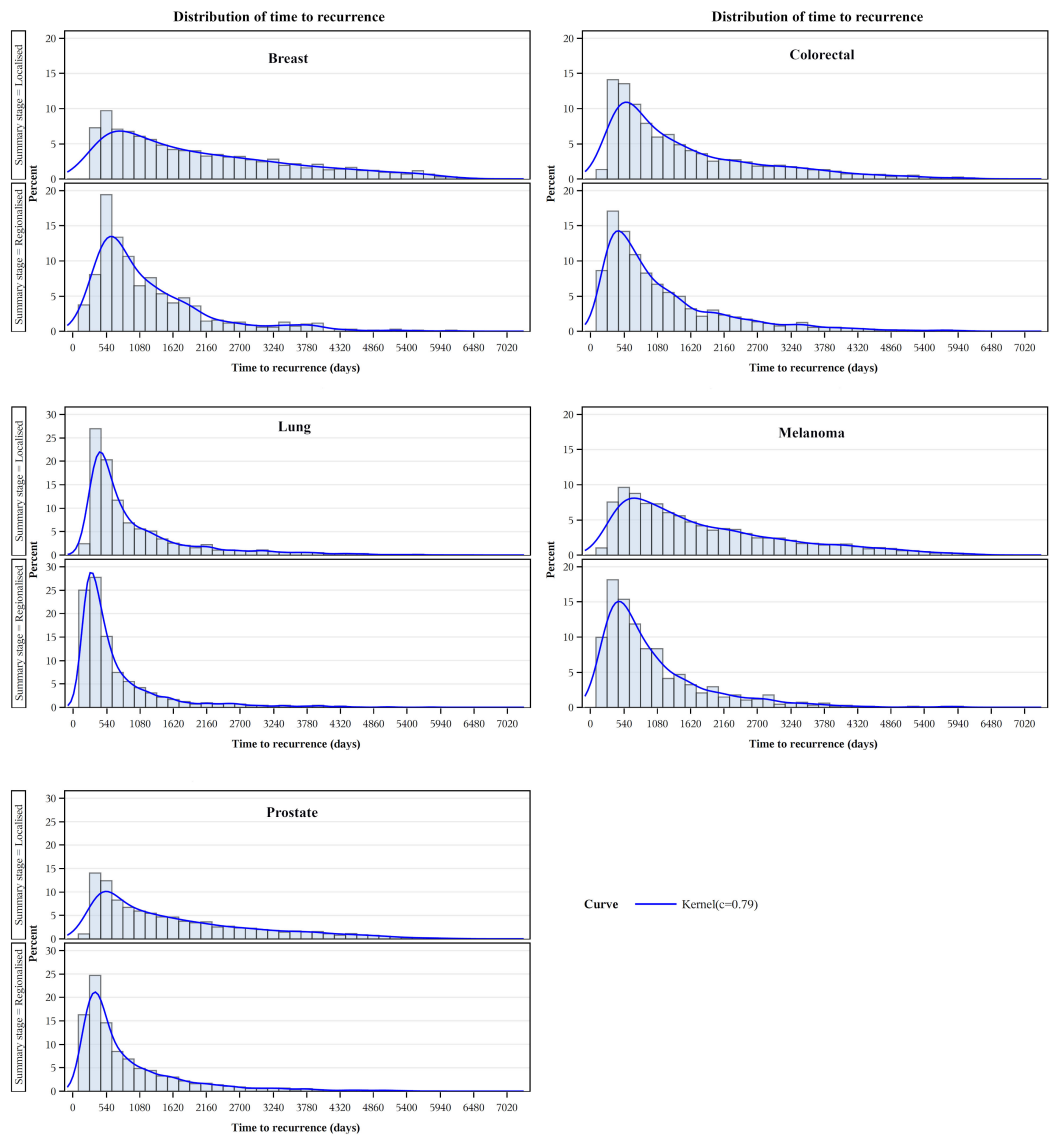
Recurrence and like event time distributions by extent of disease/summary degree of spread.

Kaplan-Meier product-limit estimates of times to recurrence or like event are shown graphically in **Figure 3**, indicating shorter times from initial diagnoses when stage was regional compared with localized. For each cancer, log rank tests indicated that these differences were unlikely to occur from chance (p<.0001). Distributions of times to recurrence or like events (histograms with 180-day bins) are shown in **Figure 4**. Cumulative Kaplan-Meier probabilities of recurrence or like events at 18 months were: for breast cancer -17% and 30% for cancers diagnosed when localized and regionalized, respectively; for colorectal cancer -corresponding probabilities of 30% and 39%; for lung cancer - corresponding probabilities of 50% and 73%; for skin (melanoma) – corresponding probabilities of 19% and 44%; and for prostate cancer – corresponding probabilities of 28% and 56%. For each cancer, peaks of the kernel curves, indicating the peak rates of recurrence and like events, generally occurred 90-100 days earlier for cancers diagnosed originally as regionalized compared with localized.

### 3.3 Case survival

**Figure 5:**
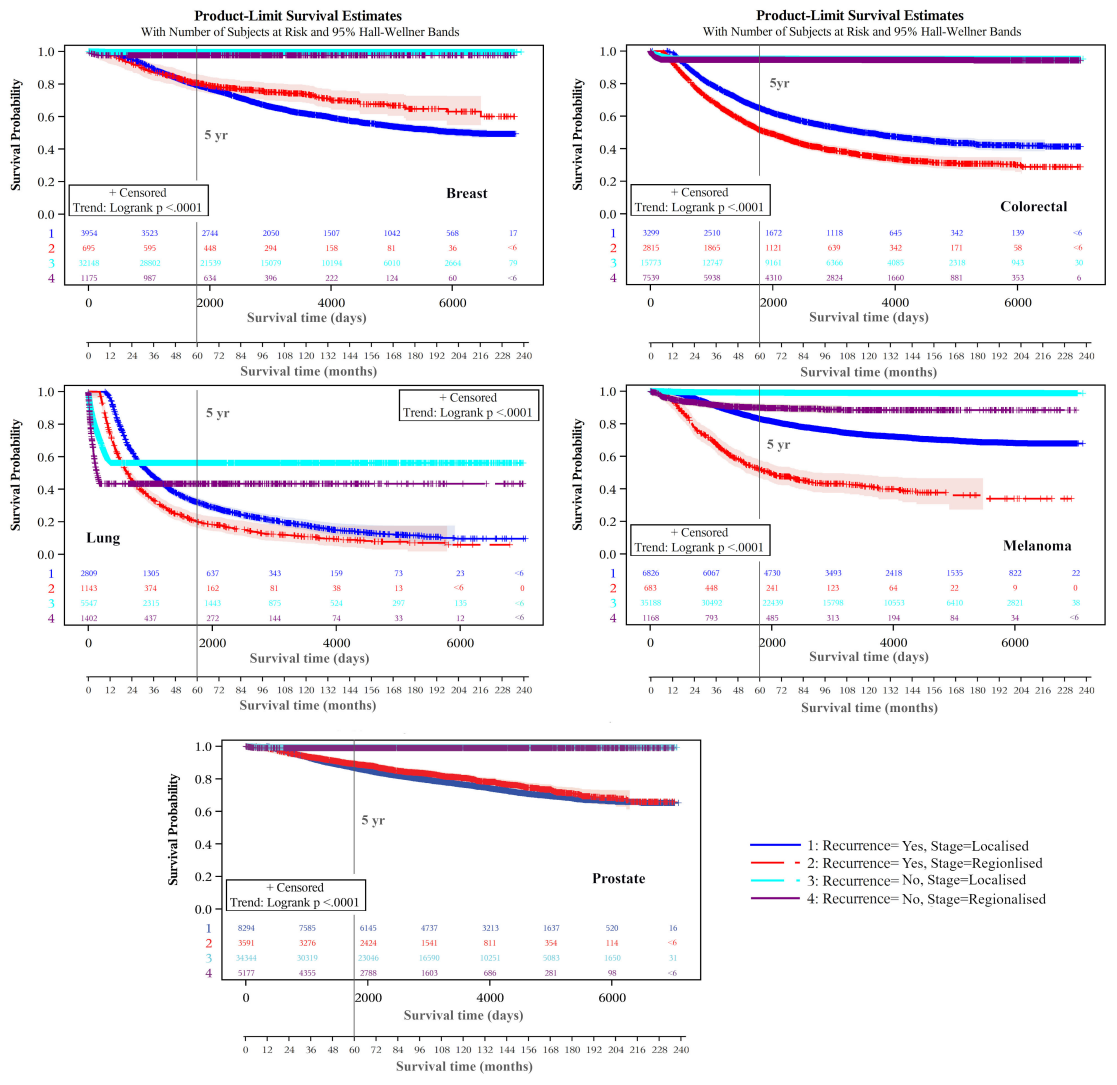
Survival from cancer as cause of death, by recurrence and like event status.

Kaplan-Meier plots of cancer-specific survival indicated higher survival over the longer term for cancers classified as not experiencing a recurrence (or like event) than those experiencing these events for each cancer type (**Figure 5**). There were earlier crossovers, however, with reverse relationships applying during the initial 16 months for skin (melanoma), lung, and colorectal cancers (note: this pattern was most marked for lung cancers, both in those diagnosed at a localized and regional stage). For breast cancers having a recurrence or like event, long-term survival was greater for regionalized than for localized cancer. For all cancers, the log rank test indicated it highly unlikely that differences seen by recurrence (and like event) history were chance events (p<.0001). Except for lower survival from localized versus regionalized breast and prostate cancers classified as having a recurrence (or like event), five- and ten-year survivals were significantly lower for regionalized cancers having such an event than for localized recurrent cancers experiencing these events (**Table 3**).

**Table 3:** Cancer-specific survival at five and ten years by summary degree of spread and indicator of recurrence or like event, major cancers diagnosed July 2001–December 2018, NSW, Australia.

Cancer	Summary Degree of Spread	5-year survival		10-year survival	
		Non-recurrent	Recurrent	Non-recurrent	Recurrent
		% (95% CI)	% (95% CI)	% (95% CI)	% (95% CI)
Breast	Localized	99.7 (99.6–99.7)	78.9 (77.6–80.1)	99.7 (99.6–99.7)	61.3 (59.7–63.0)
	Regionalized	97.6 (96.7–98.5)	80.3 (77.3–83.3)	97.6 (96.7–98.5)	73.2 (69.5–76.8)
Colorectal	Localized	95.3 (94.9–95.6)	64.3 (62.6–65.9)	95.2 (94.9–95.5)	49.3 (47.4–51.2)
	Regionalized	94.6 (94.1–95.2)	50.9 (49.0–52.8)	94.6 (94.0–95.1)	35.2 (33.2–37.2)
Lung	Localized	56.1 (54.8–57.8)	31.1 (29.3–32.9)	56.1 (54.8–57.8)	16.8 (15.2–18.4)
	Regionalized	43.3 (40.7–46.0)	19.4 (17.0–21.8)	43.3 (40.7–46.7)	10.5 (8.4–12.5)
Melanoma	Localized	99.1 (99.0–99.2)	82.8 (81.8–83.7)	98.9 (98.8–99.1)	72.9 (71.7–74.0)
	Regionalized	89.8 (87.9–91.7)	51.6 (47.7–55.6)	88.4 (86.2–90.6)	41.4 (37.1–45.8)
Prostate	Localized	99.4 (99.3–99.4)	86.3 (85.5–87.1)	99.4 (99.3–99.4)	76.1 (75.1–77.1)
	Regionalized	98.9 (98.6–99.2)	88.8 (87.8–89.9)	98.9 (98.6–99.2)	80.2 (78.6–81.7)

## 4 Discussion

Although indirect measures, the present algorithmically-derived markers of recurrence and like disruptive events showed substantial construct and predictive validity. Cancers estimated to have these events were more likely to have a diagnosis of regional than localized stage and to have shorter median times from diagnosis to recurrence. Also, the cancers classified as having these events showed earlier peaks in time-to-event distributions post-diagnosis. Cases algorithmically estimated to have a recurrence or other disruptive event had shorter long-term survival from cancer post-diagnosis than did other cases, indicating the potential predictive value of these events at a population level.

There were apparent anomalies, however, including in the early months from diagnosis (approximately 16 months) when cancer-specific survival was lower for cases not showing recurrence or like events for melanoma, lung and colon/rectum cancers (p<.0001). The reasons are partly explained by earlier cancer progression precluding case eligibility for remission and recurrence. This is supported by a more marked effect for fast progressing and lethal lung cancers than for melanomas and cancers of the colon/rectum. Another possibility could be confounding from less effective treatment practices due to unrecorded frailty and co-morbidity that reduced prospects for remission.

Notably, the small number of recurrences or like events still happening 15+ years following cancer diagnosis, noticeable for lung cancer (**Figure 3**) is the result of secondary cancers, or death from these (or lung cancer), being the only indicators of recurrence. A similar decline in survival 15+ years following diagnosis from lung cancer cause of death is also evident (**Figure 5**).

The finding of lower survival in localized compared to regionalized recurrent breast cancer may be associated with organized mammography screening, where the recurrent cases in localized breast cancer may emanate from a higher proportion of non-screen detected localized cancers being found by palpation rather than by a screening mammogram. The finding requires further investigation to rule this possibility in or out.

Overall, we consider the evidence of predictive validity is substantial and supports the potential utility of these algorithmically generated markers for population-level planning and evaluation. This could assist service planning, particularly in priority settings, for follow-up attention of high-risk sub-groups in the population. More generally, the distributions of times to recurrence or like events (**Figure 4**) provide potentially useful information for tailoring follow-up strategies to the risk of relapse (i.e. frequent for the first 2 years, with a tapering and handover to primary carer at some point, accepting that the risk never hits zero).

We also compared our results with those from cohort studies, which mostly were clinical cohort studies with case selections designed to explore clinical, molecular, or treatment relationships rather than representative estimates of prevalence at a population level. Examples included:

1. A population-based Dutch study of recurrence from operated non-metastatic breast cancer by molecular subtypes (Luminal A, Luminal B, HER2 positive and triple negative) which found at 10 years: 13% of luminal A, 23% of luminal B, 30% of HER2 positive and 27% of triple negative cancers to be recurrent, with a 10-year overall recurrence rate of 18% (over a 11.3 year median follow-up time) (32). This is higher than the estimated 12% for recurrence and like events in the present study (for ≈7-year median follow-up period). It should be noted that this Dutch cohort was not of all non-metastatic breast cancers but of those for whom surgery was performed. Also, non-metastatic cancers include those not staged (or unknown degree of spread), whereas in the present study only localized and regionalized summary stage breast cancers were analyzed, whether undergoing surgery or not.
2. Using a record-linkage algorithmic approach similar to that of the present study, an Australian study excluding metastatic and unknown stage breast cancer in the *45 and Up* cohort estimated cumulative recurrence incidence to be 12% at 66 months, compared to the present study of recurrence and like events of 8% for 60 months (23). Three probable sources of this difference are evident: (i) the algorithm used for the *45 and Up* study included as a recurrence indicator the use of chemotherapies relevant to metastatic cancer; (ii) some recurrence indicators may have been treatments for new primary cancers not yet recorded due to the cut-off date of cancer diagnoses recorded in the NSW Cancer Registry; and (iii) while the *45 and Up* study was mostly representative of the NSW population on demographic criteria, its 18% sampling fraction may indicate self-selection biases including those relating to health.
3. A UK study of recurrence for stage I-III colorectal cancers (n=1,132) (median follow-up time 4.4years), found 17% to have had a recurrence (27). Our rate of recurrence and like events was 21% (median follow- up time of 5.5 years) is comparable.
4. A US study estimated annual colorectal recurrence rates for a cohort of Stage II-III colon and rectal cancers separately for five and ten years in 1994–2003 (31). A rough estimate of overall 5-year recurrence rate can be obtained by weighting the stage II-III annual cumulative incidence rates by stage, age group, and colon or rectal primary cancer site, and multiplying the results by 5. This led to a 5-year cumulative recurrence incidence estimate of 36%, which exceeds our corresponding estimate of 25%. The disparity could reflect treatment outcome differences between study periods (1994–2003 vs. 2001–2018) or other measurement effects.
5. An Italian Environmental And Genetics in Lung Cancer Etiology (EAGLE) study reported recurrence among stage IA to IIIA surgically-treated lung cancer patients (n=768, diagnosed in 2002–2005 and followed up to the end of 2010). This found a 5-year cumulative recurrence incidence of 33%, 38%, 61%, 57% and 52% for stages IA, IB, IIA, IIB and IIIA respectively (25). Our estimate of 5-year cumulative incidence of recurrence and like events for localized lung cancer (equivalent to stages IA and IB) was 46%, and 66% for regionalized cancer (equivalent to stages IIA-IIIA). Our somewhat higher estimates are partly due to our lung cancers including non-surgically treated cases which have higher recurrence rates despite progression often precluding recurrence (25).
6. A Swedish study of cutaneous malignant melanoma investigated recurrence/progression as a combined entity and survival outcomes (n=3,554). Results indicated a 5-year recurrence/progression-free survival of 85% for stage I melanoma (cf. 89% here), 59% for stage II (which the authors classified as ‘localized’ along with stage I, cf. 62% for regionalized melanoma estimated here), and 17% for stage III (28). We note that our study covered diagnoses in 2001–2018, where results may not be applicable to the cancers diagnosed more recently in Sweden during 2015–2018 due to the treatment gains from immunotherapies and targeted therapies now offered in the adjuvant setting (e.g. nivolumab).

As these examples show, it is difficult to compare our results for recurrence and like events with the extant literature due to diversity of study designs and samples, analytic methodologies, study periods, and the cancer types, stages, and prognostic information included. We consider that further methodological development is needed for standard ongoing monitoring of recurrence rates through population-based cancer registries. Use of algorithmically-derived estimates of recurrence markers are candidates for this monitoring across Australia where data infrastructure opportunities are similar population-wide. Ideally, regular updates of clinician-informed algorithms would occur at periodic intervals to take account of treatment advances.

Excluded cancers from this study were cancers found to have unknown or distant metastatic disease spread at the time of initial diagnosis. In cases of metastatic cancer where further treatment was stopped but where the patient lives a further year or more without treatment but dies of the cancer, the algorithm would have misclassified this group as recurrent. The inclusion of metastatic cancers in future studies may be relevant, however, now that new immune checkpoint inhibitors (immunotherapy) and targeted therapies are achieving significant remissions. In particular, this may also be applicable to metastatic lung cancers, HER2-amplified breast cancers and melanoma.

A limitation of the present study is that there was no chart review ‘gold standard’ with which to compare recurrence/non-recurrence predictions. In the absence of a feasible source of such clinician-sourced data, we resorted to a survey of patients and treating clinicians of a subset of cancer cases linkable to the *45 and Up* cohort study. While this by no means was a ‘gold standard’, it proved to be useful for identifying some procedures to indicate recurrences that were initially missed in earlier versions of the algorithm.

The lack of prognostic/diagnostic data for predicting recurrence and like events, other than whether the cancer was localized or had regional spread at diagnosis, is a common problem with population-based cancer registries. Furthermore, few ostensibly recurrent cases have staging information available at the time when recurrence is detected, although limited opportunities may exist to infer (advanced) stage of recurrence from subsequent chemotherapy types, as in the cited Australian study of breast cancer (23). Overall, it remains a central limitation of developing recurrence algorithms from administrative health data where, principally for patient privacy reasons, pathology testing and radiology results and prognoses, for example, are not recorded in these health data.

Incomplete data are another issue. For example, not all chemotherapy episodes are recorded in the PBS, mainly of compounds not yet fully approved by the PBS for subsidization, but nonetheless are administered through clinical trials or compassionate access programs. It is possible that some of these unrecorded chemotherapy episodes may have resulted in recurrent cases being misclassified as non-recurrences, thereby underestimating recurrence rates.

With these caveats in mind, the main purpose of the present study was to develop and test a methodology to derive indicators of cancer recurrence and like events population-wide, by using routinely collected cancer registration and health-system usage data. Despite the drawbacks indicated above, the balance of the results supports the algorithm’s predictive and construct validity and potential value for population-wide monitoring. Strengths of the study include the application of a consistent methodology for estimating rates of recurrence and like events population-wide. While results are plausible and have construct validity, further development of this methodology and of the databases available for linkage is needed. Limitations included: the reliance on treatment disruptions to infer recurrences and like events due to the absence of direct morphology and radiology evidence for recurrences in source data at a population level; the coarse categorization of cancer types without access to population-wide data on subtypes of cancers classified by biomarkers; and the reliance on simplified staging akin to SEER summary stage, as frequently used by population-based registries.

While further work is needed to develop and validate the population-wide estimates of recurrence and like events produced algorithmically in this study, their predictive and construct validity for indicating rates of recurrence and like events and associated survival outcomes shows promise. If applied to linked cancer registry and treatment data Australia-wide, they could provide useful interim markers of recurrence and like events for population-wide monitoring and service planning.

Ongoing monitoring and updating of recurrence rates ultimately would require implementation of machine learning and/or other artificial intelligence approaches, especially in light of accelerating advances in cancer treatments where differences in recurrence rates remain a key outcome measure. Validly determined recurrences at an individual level are the foundation for this. The aim of the present study was more limited in estimating rates of recurrence and like events that disrupt event-free survival, using linked cancer registry and health-service data currently to hand.

## Data Availability

The datasets presented in this article are not readily available because these data comprise individually-linked unit record data from numerous data sources held by state and federal government health systems. Access is restricted to authorized researchers only, and its manipulation and analysis are conducted in a secured, curated research environment. Requests to access the datasets should be directed to shelley.rushton@health.nsw.gov.au.
